# Selections and Abstracts

**Published:** 1917-06

**Authors:** 


### SELECTIONS AND ABSTRACTS

### THE MEDICAL MAN AND THE SOLDIER.

Just now, with the mobilization call looked for daily, the medical practitioner who expects to serve with troops should bage cans; that the wood is neatly piled, and a sufficient quantity is stored where it will be dry should a rain come inform himself regarding the things which the soldier, the man in the ranks, demands of him aside from ordinary proficiency. These things are outside the routine of most practitioners, but upon them the life of the soldier may depend, as well as the lives of hundreds of his comrades. They are little matters, insignificant in civil practice, yet very important in the army.

The soldier wants his medical officer to be thoroughly grounded in camp sanitation, to appreciate its military importance, and to be able to teach his company officers and fellow soldiers all the details necessary to conserve their health. He knows and wants his medical officer to know that sanitation in the camp is a different proposition than in a village or city. He expects him, for instance, to locate every spot where disease carriers may breed, and to take necessary steps to prevent their development, for he is aware that it is the province of the medical officer to keep the men physically fit at all times and under all conditions, just as it is the province of the tactical officer to keep the men trained for the fight into which they are to be led.

How is the medical officer to keep the men healthy?

Sanitation will prevent most camp diseases. To get this, and keep it, the medical officer must inspect every part of the camp daily, search for foci that may lead to infection, and see that each is eliminated before it can cause trouble. This requires an intimate knowledge of many things which are not taught in colleges or mentioned in text-books, and an especial appreciation of the habits of men assembled in large numbers.

Since most medical men do not know what to look for, or where to search for it, let us make an inspection of a single company.

As we come to the cook shack, we look at the incinerator, assure ourselves that it is built and operated correctly, that the water in the suspended pails is being heated for the men to clean their mess kits after the coming meal; that all dry refuse is burned and the unburnable is prepared for the garop; that each garbage can has been scrubbed thoroughly, has a tight fitting cover; and last, but not the least important, that the ground about the shack has been carefully raked over so that any moisture may evaporate or seep away.

Upon entering the cook shack, the medical officer notes if the screening is ample to keep all flies out; if the exposed food, like bread, is stored and screened against all insects. He carefully goes over every part of each cooking utensil and piece of cutlery and tries to find remains of food, grease or other organic matter which might become bacterial culture media; he examines the serving shelf and the floor—especially the floor behind the camp range and under bags and boxes—to see if these have been scrubbed thoroughly. He questions the mess sergeant, the cooks, and the cooks' police in regard to the meal in preparation, determines by examination if the quality makes it fit for consumption, and if the rotation of the food elements is such as to give a satisfying, well-balanced diet. He carefully examines all food in storage to see that none has deteriorated since its reception in the camp. He notes the condition of the uniforms of the cooks, the shortness of their hair, the condition of the hands of all having aught to do with the manipulation of the food, and their general health. In fact, he sees that everything and everybody about the kitchen approach as closely as possible to the cleanliness of a surgical amphitheater and of the surgeon before an operation.

Passing through the messing place, notes are made relative to the condition of the ground, such as the presence of minute particles of food which might remotely become a breeding place for flies.

In the company street, the care in policing each tent is noted—the aerating of the ground of the tents, the exposure of all clothing and equipment to the sun and air, the removal of all litter. Especial attention is given to the ground where the night urinal can was placed, to see if some man has failed to use it properly.

If the camp is supplied with water from two sources—one for drinking and cooking, the other for washing—the medical officer must determine if the water in each case is taken from the proper source, and if that intended for drinking is thoroughly boiled before use.

If there is a wash trough, the medical officer must convince himself that it is cleaned daily, that it drains completely, that the adjacent ground is aerated by being raked or spaded, and that no pieces of soap are lying about.

Excepting the kitchen, nothing is as important as the policing of the latrine. If this is of a permanent type, the floor must show it was thoroughly scrubbed with soap and water, and the seat, both upper and under side, similarly treated for "crabs" (pediculi pubis), as they are called by soldiers, travel from one person to another while both are using the latrine. The medical officer also sees that the supply of the authorized toilet paper is ample and that this alone is used, that the lime is employed correctly, and that the tank is pumped out each day. If a straddle trench is built, he must satisfy himself that the construction is all that can be desired, that the screening is ample, that the fecal matter is covered completely, and that, where possible, the trench is disinfected thoroughly. The night urinal can should show evidence of careful scrubbing, be dry, and contain the proper amount of lime.

The medical officer who has a hobby in fighting flies and mosquitoes ranks high with the soldier, for these insects are very annoying about a camp and are disease carriers. Therefore, every particle of camp litter must be burned, every shallow spot that may hold stagnant water drained, every deep pool covered with oil; and, if possible, all long grass is cut or oiled—not only in the camp proper but for some distance without the guard lines. All horse litter is burned where the fumes will not come into the camp, or deeply buried.

The soldier, in fact, the tactical officer, cares little how much operative or medical skill the medical officer may have.

Both want sickness kept out of the camp and unnecessary work eliminated. Hence the medical officer who stands highest in the mind of the soldier is he who knows how to prevent sickness, how to treat each company equitably. Any other kind of medical officer is worse than useless, and should keep away from troops.—G. U. Gates, Captain, N. Y. C. A. N. G. In International Journal of Surgery.

---

### MANIC DEPRESSIVE INSANITY.

Manic-depressive insanity is one of the most prominent forms of mental disease, comprising from 12 to 25 per cent. of admissions to insane hospitals. Covering a period of eight years, with 2,264 admissions to this institution, 22.7 per cent. were of the manic-depressive type of psychoses. Characterized by groups of mental symptoms sufficiently well defined to be termed the manic, the depressive and the mixed phases of the disease. Of the etiological factors defective heredity is the most important, different authorities estimating this factor to be present in from 50 to 80 per cent. of the cases. A defective constitutional basis is often apparent in individuals previous to the onset of the psychosis. Some are peculiar, some abnormally bright, others are of an excitable disposition and subject to frequent and apparent causeless changes of mood, while a few are imbeciles from birth.

Women predominate in the disease and represent about 65 per cent. of the patients. In two-thirds of the cases the first attack occurs before 25 years of age and in less than 10 per cent. after the fortieth year. The first attack may occur as early as the tenth year and as late as the seventieth year. A patient may have many attacks throughout life with normal intervals between attacks.

These symptoms tend to arrange themselves into two large groups representing the manic and depressive phases of the disease, and a third or smaller group, the mixed phases, however, they do not always represent a clear mental picture, so that it is often impossible to draw a distinct borderline between the different phases of the disease.

The manic state is usually divided into hypomania, mania, and delirious mania.

Hypomania is the mildest form and is charcterized by increased mental activity, flight of ideas, especially noticeable in their correspondence, shifting from one subject to another and unable to bring a thought to a logical conclusion. They are usually very talkative, frequently talking about their experiences, difficulties and hardships, going into minute details, and often will distort the facts with exaggerations and misrepresentations.

Their memory, as a rule, is unimpaired and they have no genuine delusions. They bost of their own deeds and show a proportional lack of appreciation for those of others, they regard their condition as absolutely normal, believe themselves misjudged and falsely confined, usually in their opinion their relatives or friends, who were instrumental in their confinement, are the ones in need of seclusion and treatment.

These patients are usually elated, happy, cheerful and often exuberant. Sometimes they develop a pronounced humorous vein and a tendency to see the funny side of things, while at times they will be inconsiderate, saucy and rude when meeting with the least opposition.

The most striking symptom is the increased psychomotor activity. They feel compelled to be doing something all the time, such as taking long walks, the writing of a diary, numerous and voluminous letters, undertaking long journeys to renew old acquaintances, purchase property and start numerous undertakings beyond their financial and physical strength. Their actual capacity for work is much diminished. They usually lack perseverance, become negligent and apply themselves to that which is agreeable.

Many of these cases remain amiable, sociable and approachable, becoming troublesome only because of their restlessness; while others become very disagreeable and uncontrolable by their friends and relatives on account of their reckless pressure of activity. The number of hours of sleep, is, as a rule, cut short, but the actual sleep is usually profound. The appetite is good and these patients often take on weight during the attack. The course of the condition is usually uniform, improvement is gradual and often accompanied by remissions.

Manic excitement or acute mania—this, in the majority of cases, is acute in onset, in some following minor ailments while in others may be preceded by a few days of depression. This condition is characterized by great psychomotor restlessness, a pronounced flight of ideas, clouding of consciousness, disorientation and great impulsiveness.

In the phychomotor field there is great activity and excitement, becoming impossible for them to keep quiet, becoming very destructive in many instances, and irritability is very common among them so that trivial affairs will often lead to great violence and abuse. Sexual excitement manifests itself in many cases and is characterized by indecent attitudes, masturbation, exposure and demands for intercourse. Speech is often incoherent and in many cases combined with hallucinations and delusions.

The hallucinations are usually transitory, such as seeing faces on the wall, hearing voices, smelling of vapors that are liberated in their room, while a great many complain of receiving electrical shocks.

The delusions are mostly expansive, claiming they are great persons endowed with supernatural strength and power, many of them imagining themselves to be directly related to God. These delusions are often expressed with a laugh and many of them forgotten in a few moments.

Sleep, as a rule, is greatly disturbed, at times going for weeks with almost no sleep, and nutrition always suffers even though they have a good appetite. The height of the disease is usually reached in from a few days to a few weeks, genuine improvement is, as a rule, very gradual and if put under any strain during this time the flight of ideas and increased activity again become prominent symptoms. The duration varies from a few days, a few weeks, and in some many months, while a very small percentage never recover. The cases with many delusions and exacerbations of excitment last the longest.

Delirious mania—This is the extreme of the manic phase and is characterized by pronounced dreamy clouding of consciousness, intense psychomotor activity, great incoherence of speech, marked flight of ideas with numerous hallucinations and delusions. These cases are very rare and are not classed as manic-depressive insanity by a great many authorities.

The depressive forms are usually classified as simple retardation of the delusional form of depression.

The delusional form is chacterized by depreciatory delusions, especially of self-accusation and of a hypochondriacal nature in addition to the evidence of retardation to the psychomotor functions, especially those of thought and action. Consciousness is for the most part unclouded. These patients are usually well oriented, understand and answer questions correctly but always show marked retardition of thought and a tendency to revert to their depressive delusions.

Stuporous states may also develop in which almost no external impressions are apprehended and consciousness is dominated by numerous variegated and incoherent delusions and hallucinations. In this state they often will not speak at all, many imagining themselves to be greatly deformed, compelled to suffer for the wrongs of others and may complain of almost any kind of physical disability. The appetite is usually poor, often showing an aversion for food and require forced feeding; the bowels, as a rule, are very sluggish; sleep is usually very broken; skin becomes sallow and rough and they usually show a great loss of weight. The height of the condition is usually reached in a few weeks to a few months and runs a course similar in relation to time as the manic phase.

Mixed types—In these states there occur various combinations of some of the fundamental symptoms characteristic of both the manic and depressive phases of the disease. Some will show a manic phase followed by a lucid interval to be followed by a phase of depression and vice versa, while others go directly from one state into another.

In all of these cases the prognosis is guardedly unfavorable in view of the recurrences of the attack throughout the life of the individual. It is favorable for recovery from individual attacks except in 5 per cent. of the cases, from which the onset passes directly from one attack into another. At present we cannot say or judge just what the future course of a case will be, but in general, it may be said, however, that it is safe to predict frequent recurrence of attacks with lucid intervals where the psychosis manifests itself early and without external cause; on the other hand, if the first attack occurs late in life and following some external cause, there will probably be but few attacks, while in many, only the one attack.

Mental deterioration occurs in only a few cases where the attacks appear during development and are long in duration, frequent in occurrence, and of a severe nature.

There are cases which remain in the manic condition for years and are termed as chronic mania, another term, constitutional mania. Depressed cases likewise become chronic in nature and last during many years. Many of these cases in time will develop arteriosclerosis or marked senile changes leading to dementia, obliterating the original mental picture.

Treatment—Abstinence from alcohol; should not marry. Treatment during manic attacks is always more satisfactory in some sanitarium or hospital. For the manic phase, prolonged hot baths, some using sedatives, hyoscine being highly recommended by some authorities. Depressed cases usually do the best with some rest treatment, nourishment carefully looked after, precautions to prevent suicide, treating the insomnia by the usual methods.

Cases representing the various types of the manic-depressive psychoses were presented.—Dr. W. S. Wentzel, M.D., Indianapolis Medical Journal.

---

### MEDICAL GYNECOLOGY.

We are frequently charged by the laity and occasionally by our colleagues with an undue and unwarranted lust for operative surgery—with a too hasty resort to the knife in some cases where careful study and patient treatment might have averted such heroic treatment. Without attempting to controvert this charge or to justify the work of the conscientious surgeon, I wish to consider a few gynecological conditions which may properly be treated by non-operative means, but which are often referred to the operative surgeon, too frequently with the "operation suggestion" firmly implanted in their minds, by the very men who charge us with over activity. I am sure the awkward position of having patients come all prepared for operation, which an examination proved to be unnecassary, is not an unusual one to my hearers. This paper is my own answer to the question, "What gynecological conditions are amenable to non-operative measures?" and "What non-operative measures are applicable to such cases?"

Gynecological patients usually consult their family physician and it is he who has the first opportunity to make the diagnosis which shall put them on local, palliative or radical operative treatment. For his guidance I would suggest a simple classification of his findings into congenital defects or deformities, neoplasms, infections, traumatism, incurred during labor and the sequelae of labor abortion. From this list I would at once strike off as unsuitable for local treatment in its commonly accepted meaning, all new growths, tumors of any kind or location. Even though nothing more than a urethral caruncle or small uterine polyp extruding from the external os be found, with which he may be entirely competent to cope, yet his treatment of these conditions must be considered surgical and not within the scope of this paper.

Regarding congenial deformities and foetal remains, no such sweeping statement can be made. The sterility and dysmenorrhoea due to infantile uterus may often be relieved by medical and local treatment, while artesia at any point of the genital canal, septa of the vagina or uterus, or absence of any of the component organs are absolutely unsuited to these methods. Young or unmarried women suffering from these conditions are often subjected to gross mal-practice because of the aversion physiciaans feel to examining such cases. The most classical case of atresia of the vagina which I have encountered had dragged out a miserable existence from puberty to marriage, with constantly increasing pelvic pain and a slowly developing enlargement in the pelvis, until the perineum was stretched as with the foetal head and the rectum was almost entirely obstructed before an examination disclosed the real nature of her distress. We must not allow our modesty to interfere with our duty. As for the patient, such a sufferer has no modesty left; her sole thought is to be relieved.

Infections from the standpoint of treatment, must be sub-divided into acute and chronic types, the nature of the infecting organism, whether the gonococcus, streptococcus, the colon group or what not, and also the point of attack must be considered. Vaginal, urethral or bladder infections and puerperal infections of the uterus, almost a crime in our day, should be met with prompt and vigorous resistance. Acute infections of the tubes, pelvic peritoneum and cellular tissues are best treated by palliative measures, watchful waiting, not unmindful of vaginal douching and vaginal puncture. Many of these cases recover by resolution, while many more, after the storm, must be treated by radical or conservative surgery. It is in this latter class of cases that much of the abuse of local treatments is seen. The family physician must know the limitations of palliative treatment and must not subject these patients to months or years of tampons, douches and chronic invalidism.

Injuries from childbirth, neglected or unsuccessfully repaired at the time, which destroy supporting structures or leave eroded surfaces for the absorption of infection, are not properly cases for palliative treatment and should only be so managed when there exists definite contraindications for radical treatment. Other sequelae of labor, of which subinvolution is the most frequent and has the greatest morbidity, if uncomplicated by injury, may usually be best treated by palliative means.

The management of these cases, then, hinges on accurate diagnosis, good judgment, and a knowledgment of the possibilities and limitations of therapeutic measures. I shall make no pretense to a comprehensive consideration of the subject but wish rather to bring to your attention a few of the commonly encountered conditions which I believe to be properly and often best treated by local means, together with some methods of treatment which I have found extremely useful but which have been more or less frowned upon by the authorities, and somewhat feared, doubted, or ignored by the profession.

Congenital deformities and diseases are often difficult of detection because so often encountered in virgins. I would emphasize the value of the bimanual examination per rectum in these cases especially, although it is often of great service in parous women. Local treatment of non-parous women is limited to those in whom the hymen and vagina will permit instrumentation. In the congenital classification I include those abnormalities which have existed from birth, or which are first evidenced at puberty or after marriage and which are not neoplastic, traumatic or infectious in origin.

Infantile uterus, when possible, is best treated by non-operative means. In addition to the usual hygienic and tonic measures, the use of graduated uterine dilators, the intrauterine application of 95 per cent. phenol or iodine, and if possible, after some progress, the use of a stem pessary, which may be left in situ with impunity for many months, together with the careful administration of small doses of thyroid extract, one-fourth or one-half grain twice daily, has been more fruitful of result in my experience than the usual curettage. It is well known that thyroid extract has a selective action on the generative organs, stimulating them to normal activity. I shall refer to its use in other conditions later.

Amenorrhoea when not due to the great physiological cause, pregnancy, or to some physical obstruction, may well be treated by much the same methods and with excellent results. Of late, I have used corpus luteum instead of thyroid extract in these cases and with much satisfaction. In the strumous type, however, thyroid extract always has a marked effect for good. Curettage does these patients little or no good and its results are almost always temporary. Depending on some obscure fault, local stimulation, together with our feeble, groping opotherapy, seems, and is far more rational than curettage. Yet we have many of these cases referred for curettage. Actual physical obstruction is about the only indication for operation in these cases.

Dysmenorrhoea is one of the most frequent complaints the physician hears, and it merits all the attention that its victim insists on, for aside from the danger of drug habits, whether as a cause or effect, it is so often associated with cystic disease of the ovaries as to be considered more than a mere coincidence. I am firmly of the opinion that many ovarian cysts are due to back pressure from a spasmodic dysmenorrhoea.

Now we are offered as our defensive weapon, dilatation and curettage, and who of us has not been disappointed in its results in, I may safely say, the majority of cases? Having ruled out, by careful examination, cystic disease of the ovaries, tubal disease, varicosities of the broad ligaments, etc., i. e., having arrived at a positive indication for curettage, I contend that the use of graduated dilators where necessary, the intrauterine application of phenol and iodine, followed by the introduction of a stem pessary, and in some cases, the use of very small doses of thyroid extract, will accomplish results of a far more lasting and satisfactory character than the orthodox treatment with the curette.

The stem pessary is looked upon with fear by many physicians. I have used them for many years, introducing them at the office under fairly aseptic technique, and have left them in place for as long as twenty months, with none but good results. There are a few patients with a positive indication for them where the vagina is so small or the cervix so tight as to preclude their use except with an anesthetic. A curious fact has been that when introduced after dilatation under anesthesia, they have invariably been expelled within a few days, even though I have tried to insure their retention by a suture of kangaroo tendon through the cervix. This shows that our rapid dilatation lasts for a short time, that the cervix does not shut down enough to hold the pessary, but a return of the old symptom proves that it is not for long. In these cases I have usually been able to replace the pessary before contracture took place. Here again, I wish to emphasize the action of thyroid extract on the pelvic organs. Do not forget it in the treatment of amenorrhoea, dysmenorrhoea and sterility.

Endometritis, leucorrhoea, menorrhagia, dysmenorrhoea, these are almost synonymous terms. In cases where the treatment can be carried out, I have rarely administered an anesthetic for curettage alone and I submit that I have had more complete and lasting results than from operative treatment. Dr. Charles A. L. Reed was the first to recommend the use of phenol as a local application to the endometrium so far as I know, and since reading his work some ten years ago, I have used this treatment in endometritis almost to the exclusion of curettage or in connection with it where anesthetic had to be administered for some other purpose. Graduated sounds are used when necessary, and the uterine cavity swabbed out with 95 per cent. phenol, or better, where possible, one-half inch selvage tape saturated with it, is packed into the cavity and is left for twenty-four hours, unless expelled by uterine contraction. A dossel of cotton saturated with alcohol is placed behind the cervix to neutralize the overflow. Appropriate tampons may be used in the vagina at the same sitting and the whole arranged so as to be easily pulled out in twenty-four hours. A hot saline douche should be used immediately after the withdrawal of the tampoon and packing, and in most cases can be used to advantage every night until the treatment is repeated, which is ordinarily on the fourth or fifth day. A wide experience with these measures enables me to say that they may be carried out at one's office without infection, that there is no danger of toxic symptoms from the phenol, and that the results are excellent, better as a rule than simple curettage in like conditions.

Retroversion is a condition which may be congenital or at least, discovered very early in life with no history to explain acquirements, or it may be acquired. In my operative experience it is unusual to find the fundus adherent except in those cases where a pelvic cellulitis has matted all the structures together, although we are quite apt to say it is adherent in cases in which we are unable to reposit it except by abdominal section. It may exist without symptoms and be discovered accidently. I rarely apprize a patient of its presence unless she is conscious of symptoms which may be properly attributed to it. It is like a movable kidney—what she don't know, won't hurt her. On the other hand, I can not agree with some gynecologists who contend that retroversion per se, causes no symptoms but that an entirely independent lesion in the tubes or ovaries is responsible for the symptoms. While the pain or heaviness or dragging may come from the latter, I am of the opinion that the tortion of the broad ligaments with the resulting interference with the pelvic circulation is the cause of most of the pelvic symptoms and is therefore part of retroversion. In the apparently adherent fundus, a thickening of the viginal ring, the interposition of a heavy sigmoid, or the incarceration of the fundus between the utero-sacral ligaments is probably at fault and before deciding that a given fundus can not be replaced without operation, let me urge a trial of the knee-chest position and bimanual manipulation with one finger in the rectum. One other method remains before resorting to operation, and I know I shall be severely criticized for mentioning it, but I can only plead my own experience, not inconsiderable in this class of practice. I often pry up such a fundus with the uterine sound, taking it out and bending it more nearly to a right angle as the fundus comes up and continuing until I can grasp the fundus through the abdominal wall. Is there danger of puncturing the uterus? Not in the hands of anyone capable of undertaking any such work. One can tell how much resistance he is encountering, as a rule, very little, and it need only be raised till the abdominal hand can catch it. I prefer this to the uterine repositor made for the purpose, for this is quite large and hard to introduce and working with a screw adjustment, one can not judge so accurately how much force he is using and might well injure the uterine wall.

Having placed the fundus in a position well to the front, with the cervix pressed back into the hollow of the sacrum, the next question is how to maintain this position until it accustoms itself to its normal position and until the supporting structures undergo involution. In cases where there is considerable bogginess or inflammatory swelling with tenderness, for a short time wool tampons with dehydrant, antiseptics and antiphlogistics, such as combinations of ichthyol, iodine and glycerine, may be used with good effects. This treatment requires daily or bi-daily visits to the physician's office and should be superceded very soon for the support that can usually be adopted at once, the pessary. My experience would indicate that physicians are not sufficiently familiar with the uses of this useful device. The younger men, especially, seem to be taught all about the surgery of this condition but very little about its non-surgical treatment. Perhaps if they were told that there are some fifty different methods or surgical relief for retroversion and that forty-nine professors are making fun of the method their teacher has honored with his name, they might conclude that the surgical treatment of this condition is not entirely satisfactory and that the fifty-first method might make their patient as comfortable as any of the others. The bogie of ulceration from pressure with ultimate sequestration within the tissues to often mentioned in the text-books, does not obtain at the present time when patients are more generally schooled in matters of health.

With a knowledge of the proper function of the pessary, to elongate the vagina and thus pull the vaginal ring back into the hollow of the sacrum, allowing the fundus to fall to the front, instead of expecting it to replace a retroverted uterus, with ordinary skill and tact in selecting the proper size and bending it to fit the case in hand, the pessary is a most useful accessory to gynecological work. There are a few cases where a pessary with a very slight angle to its posterior curve may be used to elongate the vagina before it is possible to reposit the uterus, but these are rare.

Is pessary treatment of more than temporary relief? Yes, in many cases. In both parous and non-parous patients, where there are no adhesions and no serious injuries to the supports, the utero-sacral ligaments will shrink up and draw the cervix back into the hollow of the sacrum, the round ligaments, relieved of the stretch to which they have been subjected will take up and tilt the fundus to the front and the twist in the broad ligaments having been relieved and the normal circulation restored, the boggy fundus with its sensitiveness and discharge and chronic infection, undergoes resolution and these results are just as permanent and more anatomic than where operative means have been employed.

One other common condition that may often be more satisfactorily managed by means other than operative is that monument to bad obstetrics, so prolific or morbidity, subinvolution. My experience has been that old, aggravated cases of this type, when existing without lacerations, are not cured, or at least their recovery takes months or years after the usual operative treatment, curettage, with or without plastic reduction of the cervix. In these cases, the use of the pessary or iodine applied as before suggested, and in these cases the cervix is usually sufficiently open to permit packing the cavity with gauze, will give better results than a single curettage insure a more perfect result when operatoon is undertaken.

The foregoing constituting the large percentage of our every-day gynecological complaints, are the ones in which I have found office treatment of the greatest service. Results, if forthcoming in this way, are usually attained in from six to ten treatments. The phenol treatment is heroic and I always advise the patient beforehand that if no improvement is experienced within that number, more radical means must be adopted. I have as little sympathy with protracted courses of treatment over month or years as with the exclusion of all office treatment.

I am well aware that some of my contentions will meet with opposition; that they are frowned upon by many of the authorities. I can only reply that this paper is based exclusively on my own experience, entirely ignoring the authorities; that considerable office instrumentation may be done without infection by exercising ordinary precaution; and that in quite an extensive practice in this line of work, I have secured results that merit my conclusions.—New York State Journal of Medicine.

---

### SPECIFIC TREATMENT IN PULMONARY TUBERCULOSIS.

**By Gilbert T. Brown, M.D., Dayton, Ohio.**

In presenting my clinical experiences and results in the treatment of pulmonary and other forms of tuberculosis during the last twelve years, it is with the belief that the methods of treatment which were employed will be helpful to those who still depend upon the usual hygienic and dietetic methods alone, as I had done myself during the preceding years of my practice.

Like others I found that the majority of tuberculosis patients are unwilling and many of them entirely unable to seek their recoveries away from home under the care of specialists or in private sanatoria, and also that the commonly practiced methods are to say the least but rarely successful. Indeed, I do not remember a single case of the disease in which I accomplished a complete clinical recovery prior to my inclusion of specific remedies in their treatment.

In 1904 my attention was attracted to the watery extract of tubercle bacilli, as used by Dr. Karl von Ruck in his sanatorium for tuberculosis in Asheville, N. C. I have used this preparation upon most of the patients under consideration. More recently I have also used his vaccine against tuberculosis, and the results obtained and to be related will make apparent the benefits which my patients derived.

I am in a position to report upon the results in fifty-two cases which were treated by me since 1904, and in so far as the patients are still living they have been kept under observation to the present time.

Of these cases twenty-two, or 42.3 per cent., were in a comparatively early stage; thirteen, or 25 per cent., in a moderately advanced stage; and seventeen, or 32.5 per cent., were in the far advanced stage of the disease, at the time when the specific treatment was first employed.

Not desiring to detain you with the details of the case histories of these patients, and yet wishing to give you some data of their condition when they came under my care and on their discharge. I give essentials especially of the most important features which were observed.

The nutrition in tuberculosis often suffers at a quite early period, and all excepting two of my patients had lost weight which varied from five to fifty pounds, averaging about twenty-one pounds.

After discontinuing treatment forty-five patients showed gains in weight varying from five to forty-five pounds and averaging about eighteen pounds; five patients had either shown no gain or had continued to lose.

It seems to me that the improvement noted in nutrition is in itself indicative that the patients profited by the dietetic regimen which was followed; the specific treatment favored a better metabolism and improved the patients' appetite, and assimilation became so strikingly apparent in certain instances that there could be no doubt of the relation, because their appetite improved promptly when for weeks and months there had been no desire for food, and the steady decline in weight ceased and was followed by prompt and steady increase. Four patients with apparently sever gastric complications, and whose stomachs rejected almost everything and could not digest but little, were able to digest full, generous meals at the end of the first month without the aid of other therapeutic measures.

Fever was absent in only five or 9.6 per cent. of my patients. In twenty-eight it was moderate in degree, not exceeding a maximum of 101.5 degrees. In nineteen it ranged higher, especially in the far-advanced cases; maxima of 102 degrees were frequently noted; and in several instances the afternoon temperature was 103 degrees, and in one case 104 degrees Farenheit.

On discontinuing the treatment the temperature was normal in thirty-six, or 69.2 per cent.; it did not exceed 100 degrees F. in eight, or 15 per cent. In the other cases, in which the type of the pulmonary disease was as a rule pneumonic and mixed infection appeared to dominate the clinical course, the fever continued unabated, or was but slightly less than on admission.

Cough and expectoration was as yet absent in seven, or

11.7 per cent., of my early-stage cases. These symptoms were slight in twenty-three, or 45 per cent.; moderate in degree in fourteen, or 24.9 per cent.; and severe in eight or 15.6 per cent. Tubercle bacilli were found in the sputum in twenty-nine, or 58.8 per cent.

On discontinuance of specific treatment there was no cough or expectoration in twenty-eight, or 53.8 per cent.; only slight in fourteen, or 27.4 per cent.; moderate in six, or 11.7 per cent.; and severe in four, or 7.8 per cent. Tubercle bacilli were absent in thirty-seven, or 71.1 per cent., and were found in fifteen, or 28.8 per cent.

Mixed infections. Other pathogenic microorganisms were found in twenty-five, and in twelve, or nearly half, of them their relation to the clinical course appeared probable in that the type of the fever was hectic, reaching maxima of 102 degrees and over. The organism in relation was as a rule a pneumococcus; in a few cases the influenza bacillus was found. In several of these cases the associated infection yielded to stock vaccines, of which I availed myself in more recent years, and which would probably have prevented several failures in my earlier cases.

Hemoptysis. Pulmonary hemorrhages had occurred prior to treatment in several of my patients; none occurred during the administration of watery extract or of the vaccine, although marked focal reactions occurred repeatedly in the affected lungs in almost all cases. In one patient, treated in 1913, slight hemoptysis, amounting to less than one ounce, occurred after the treatment was discontinued, which caused its resumption, and the patient has since continued in good health.

Night sweats. Before being treated, night sweats had occurred regularly in six and occasionally in eleven of the patients. This symptom was still present on discharge, in four of whom the treatment had also failed to cause other improvement.

Tuberculosis complications. Tuberulosis of the larynx of a marked degree was recorded in eight cases, four of which showed ulceration and belonged to the far-advanced class of progressive phthisis. One of them treated in 1905 recovered, and is still in good health; of four cases in which the laryngeal affection was well marked but had not progressed to a stage of ulceration, two treated in 1907 recovered and are still in good health. In numerous other instances, slighter symptoms, such as hoarseness, were present or focal reactions occurred in the larynx, but owing to my limited experience in laryngology I could not make a positive diagnosis. I therefore leave them out of consideration, but if those cases represented tuberulosis of the larynx in an early stage, recovery followed in all of them.

Tuberculosis of abdominal organs, including intestines, genitalia, and peritoneum, was diagnosed in seven cases, six females and one male. An interesting feature in all of them was the occurrence of the focal reactions which manifested themselves by increase of the local symptoms. In two of these patients treated in 1906 and 1907, and in whom tuberulosis of the intestine and plevic organs was a complication of the pulmonary disease, complete recovery followed, and both are well at this time. Another case treated two years ago, with parametritis on the left side and marked focal reaction in the lower part of abdomen and the rectum, was greatly improved; her pulmonary symptoms having disappeared she discontinued treatment. She has continued in a fair state of health. A fifth case treated in 1914 had previously been operated upon for rectal stricture; she had a rectovaginal fistula. A Wassermann reaction was positive, and positive focal reactions were likewise observed on the administration of the von Ruck vaccine. Another operation became necessary on account of the rectal stricture, and succinimide of mercury was thereafter administered for nearly a year. This patient recovered from her pulmonary tuberculosis, and recent reports indicate improvement of the rectal affection. The other two patients succumbed to their disease.

Tuberculosis of bones and joints was present in three of the cases. One case of tuberculosis of the knee-joint treated in 1907 in a far-advanced stage of phthisis recovered from both his joint and pulmonary disease and has continued in good health since.

Tuberculosis of the vertebra was found in two cases; in one the first dorsal vertebra was involved, but the pulmonary disease was comparatively slight; this patient also had pelvic tuberculosis and probably also tubercle in the intestine; marked focal reactions followed the administration of the vaccine in its vertebral lesion and were attended with sharp attacks of diarrhea. This patient has also apparently recovered. The other case of spondylitis of first and second dorsal vertebra had on active lesions in the lungs, and the focal reactions, which were well marked, were limited to the vertebrae. The reactions grew less and eventually ceased. This patient is still under observation, having markedly improved. He is wearing a brace, and the spinal curvature is becoming markedly less.

Non-tuberculous complications were observed in a considerable number of the patients under consideration, especially in the moderately edvanced and far-advanced stage. Several such complications were at times present in the same case, and the pulmonary disease was further complicated by extra-pulmonary tuberculous affection and by mixed infections to which I have already referred. In other cases anemia, albuminuria—with or without casts—gastric catarrh, and gastroptosis, cholecystsitis, malaria, chronic nephritis, and syphilis were recorded on their admission. Altogether there were fifty instances of non-tuberculous affections coexisting, which were treated with success in twenty-three instances; twelve other cases improved, and in the remainder no material improvement could be observed.

Considering this clinical material as a whole it is evident that a considerable number offered formidable obstacles, both on account of the advanced stage of the pulmonary disease and of the complications that were present.

A tabulation of the clinical results observed shows that recovery occurred in all of the twenty-two patients who were in a comparatively early stage. Seven of the thirteen moderately advanced cases of phthisis recovered; three cases were improved sufficiently that the disease could be considered as arrested; three others were improved. In the seventeen patients of the last stage of phthisis only two recoveries occurred. The disease was arrested in three and improvement was noted in three others. No result at all was obtained in nine cases. This totals for all stages thirty-one recoveries, or 59.6 per cent.; six arrestments, or 11.5 per cent.; with lesser degrees of improvement in six others, including the coexisting complications.

Of great interest appears to me the permanency of the clinical results. All except one of the patients here considered have been traced, and most of them have been under more or less continued observation. The results as determined at the present time are as follows:

Early stage, 22 cases. All are living; eighteen are in good and three in a fair state of health, without recurrence of their tuberculosis; one could not be traced, but it was a mild case and the recovery appeared complete; it is not probable that a relapse occurred.

Six patients who were treated in this stage were dismissed ten to twelve years ago.

Twelve patients were dismissed since 1910.

Moderately advanced, 13 cases. All are living; ten are in good and three patients in a fair state of health, without relapse since their dismissal.

Nine patients were dismissed ten to twelve years ago.

Four patients were dismissed since 1910.

Far-advanced stage, 17 cases. Six are living; four in good health, and two patients have continued in fair health without relapse since their discharge. In seven patients, after having improved, experienced a relapse; all have died since their treatment; one died of acute nephritis seven years after her complete recovery, during which she enjoyed good health and had gone through two pregnancies, and both children so far as I am informed are living and in good health. Of the six patients now living four were treated ten to twelve years ago, one eight years ago, and the other was dismissed during the present year.

Conclusion. With only ten deaths from tuberculosis, all of which occurred in instances of far-advanced consumption, and the remainder living in good or fair health without relapse after their apparent recoveries and after periods up to twelve years, I feel justified in concluding that great aid from specific remedies applied has accrued to the forty-two patients, forty-one of whom are now living in good and fair states of health. Without specific treatment added to the ordinary hygienic and dietetic methods my results in the treatment of tuberculosis prior to its adoption were practically negative.

My experiences also show that specific remedies can be applied to general practitioners with safety and with approximately equal success as is obtained by specialists, and this is undoubtedly true of the use of Dr. von Ruck's preparations, which are the only ones with which I am practically familiar.

The watery extract which I employed prior to the introduction of his vaccine has produced equally good results, and some of my most gratifying successes in the advanced cases of phthisis were obtained with it. The vaccine appears, however, to me to be a more powerful immunizing agent, and in early-stage cases especially the improvement observed by me appeared more rapid and an apparent recovery was effected in considerably shorter time.

The methods of preparing the watery extract of tubercle bacilli and that for the preparation of the vaccine against tuberculosis were given in their respective announcements.

The formula for the watery extract of tubercle bacilli is given in a paper read before the American Climatological Association, which appeared in its Transaction for 1897, and also in the Therapeutic Gazette of the same year, page 388.

The preparation of the vaccine is given in a report from von Ruck Research Laboratory, June 1, 1912, and in vol. 82, 1912, p. 369, of the Medical Record. Sufficient details are supplied in either case to enable any well-equipped laboratory to manufacture an identical preparation.

The watery extract represents a solution of extractives from tubercle bacilli in water after the previous extraction of their lipoids with ether and alcohol.

The vaccine is represented by all body extractives and in definite proportion of the several proteids, albumin, and lipoids. It is balanced that the most important immunizing body substances of the bacilli are present in larger amounts, whereas the lipoids are reduced sufficiently to remove the danger of local necrosis at the site of subcutaneous injection.

The advantages of the vaccine are considered to depend upon the presence of all the immunizing body substances of tubercle bacilli in solution without chemical or physical injury in the course of their extraction, and in such proportions that much larger doses can be administered than of a tubercle bacilli emulsion. Being a solution of the bacillary body substances, its action is certain and uniform and occurs with a promptness sufficient to insure a complete bacteriolytic immunity within a few days after the administration of a single dose for the respective age of the individual, features which the authors consider especially essential for prophylactic vaccination in order that the latter shall be practicable, reliable, and simple, the same as is vaccination against smallpox or typhoid fever.

In order to prevent self-treatment and the use of the preparation by incompetent persons, they are not supplied to the trade and must be ordered directly from the laboratory by physicians.

The technique of administering the watery extract of tubercle bacilli and the vaccine for the treatment of tuberculosis is the same. Physicians receive with their first order general directions in regard to dosage and the selection of the cases; and I have found the Drs. von Ruck extremely courteous and ready to consider and answer by letter any questions that may arise, with the view of aiding those less experienced in successfully treating their tuberculous patients at home.

Inasmuch as the degree of spontaneously acquired immunity in tuberculous patients varies, the degree of sensitiveness and liability to reaction does so likewise. To prevent severe reactions the beginning dose should be minimal, and is from 0.05 to 0.1 Cc. of the full strength of the remedy. On request a 10 per cent. solution of both preparations is supplied by the laboratory, and directions are given for their preparation, which is simple, and requires the use of boiled water containing 0.5 per cent. carbolic acid in proportion to one part of the remedy and nine parts of the water.

Injections are made subcutaneously, and the locality must be changed frequently in order to prevent the neutralization of the dose by the occurrence of a local immunity greater than that of the blood and of distant tissues.

The increase of doses depends upon observed general and focal reactions; so long as these occur no increase is permissible, and if severe, the dose should be reduced. The interval between doses is five days providing no reactions follow or when observed if the symptoms and signs disappear within forty-eight hours after administration. The subsequent increase of doses has for its object the continuance of periodical specific stimulation of tuberculous lesions and the increase of the general immunity. For the size of subsequent doses, individualization is necessary. As a rule, and especially in the early part of treatment, and increase of 0.1 Cc. is sufficient. There is no maximal dose, but it is not often necessary to exceed 1Cc., especially if the site of the injection is varied. Intravenous administration is recommended when reactions to 1 Cc. fail; the intravenous dose should not exceed one-fourth of the dose which failed to cause reaction.

For prophylactic administration in instances of clinically non-tuberculous persons, and such who have been exposed to infection who are free from fever, and in good nutrition, a single maximal dose may be given, which in size varies according to age and weight. For children up to one year of age the dose is 0.1 Cc., for adults weighing in the neighborhood of 150 pounds it is 1 Cc. In cases which present evidence of tuberculosis the procedure to be followed is the same as outlined above for treatment.—The Therapeutic Gazette.
Ohio. The Therapeutic Gazette.

